# We need to stop female genital mutilation!

**DOI:** 10.1186/s12978-016-0131-2

**Published:** 2016-04-18

**Authors:** José M. Belizán, Suellen Miller, Natasha Salaria

**Affiliations:** Institute for Clinical Effectiveness and Health Policy (IECS), Buenos Aires, Argentina; University of California, Sacramento, USA; BioMed Central, London, UK

Over the next decade around 30 million girls under age 15 are at risk of FGM/C. Given that there is no physical benefit for the girls and acknowledging that FGM/C involves physical, psychological, social and reproductive harm, we, along with major international and national governmental and non-governmental organizations find FGM/C a severe violation of human rights [1].

We must encourage vigorous action among health providers, civil society, women’s organizations, funders, international agencies, international and national courts of justice, global and religious leaders, and governments to change this unacceptable practice.

It is painful to see that parents and families are imposing this practice on young girls including newborns. They are imposing their wills on the bodies of young girls, who have no chance to participate in a decision that affects their health, safety, and lives, thus violating their human rights.

While in some countries legislation has been attempted to stop FGM/C, wealthy families often avoid legislation by taking girls to another country or by utilizing licensed medical personnel. If a procedure violates a girl’s human rights, is it better in the hands of a medical provider? While not denying that having medical personnel perform FGM/C is “harm reduction, ” and increases the safety of the physical procedure, for example, decreasing the risk of infection, the procedure is still a human rights violation. Should medical professionals be involved in performing FGM/C in any instance? Should national and international professional organizations, universities, and governments protect girls and women by having guidelines that strongly recommend against FGM/C? Would religious leaders be willing to make clear statements condemning this practice?

While FGM/C is defined as cutting the genitals for “non-medical purposes” a common obstetric practice, **episiotomy**, when performed without clinical indications, can be categorized as FGM/C [2]. Despite evidence disseminated more than 20 years ago that routine episiotomy was unnecessary, and should be abandoned; the practice has continuing high incidence, with greater than 90 % of nulliparous women receiving episiotomy in facility births in many middle and low income countries [3–5]. Of great concern is the fact that low-income countries, which are making great efforts to increase institutional deliveries, are performing higher rates of unnecessary episiotomies. Increasing rates of facility births are concurrent with increasing rates of unnecessary episiotomies, despite clinical practice guidelines that recommend against the practice.

How we can explain that there is so little research looking at interventions that can decrease FGM/C and episiotomy? National and international funds, research institutions and, multi-disciplinary teams should be dedicated to develop and test interventions that successfully lead to communities abandoning FGM/C and to practitioners abandoning unnecessary episiotomies. Different research methodologies should be used, as random assignment to control groups or “usual and customary practice” could be an ethical concern. However, study designs such as before and after, cluster randomization, time-series, step-wedge, and similar are warranted. It would be most desirable to have transdisciplinary teams, including social scientists, epidemiologists, health providers, women’s organizations, communities’ representatives and politicians to develop, implement, and evaluate the interventions.

*Reproductive Health* is eager to contribute to decreasing FGM/C and episiotomies by publishing a variety of research and information related to FGM/C. Submissions can be in a variety of formats, such as personal testimonies - i.e. a woman describing her own experience, case studies, descriptions of programs, and reports of interventions that have been tested and shown to decrease FGM/C or episiotomies. We will encourage publication about these programs at local, regional or hospital level and also changes over time of prevalence/ incidence. We will also publish protocols or information on initiatives that aim to develop and test interventions that could decrease FGM/C and episiotomy. Qualitative studies from affected women, providers, traditional health workers, political and religious leaders will be especially welcome. These contributions will be published in the form of an FGM/C-related themed series, brought together in a collection for easy and open access to the research and highlighted on the journal homepage.

We at *Reproductive Health* are committed to helping communities, researchers, practitioners in the fight to abandon FGM/C, and, especially committed to helping women and girls have the opportunity to live an intact life, free of mutilation and the negative consequences of FGM/C.
